# Defining the genes for the final steps in biosynthesis of the complex polyketide antibiotic mupirocin by *Pseudomonas fluorescens* NCIMB10586

**DOI:** 10.1038/s41598-018-38038-9

**Published:** 2019-02-07

**Authors:** Jack A. Connolly, Amber Wilson, Malgorzata Macioszek, Zhongshu Song, Luoyi Wang, Hadi H. Mohammad, Mukul Yadav, Maura di Martino, Claire E. Miller, Joanne Hothersall, Anthony S. Haines, Elton R. Stephens, Matthew P. Crump, Christine L. Willis, Thomas J. Simpson, Peter J. Winn, Christopher M. Thomas

**Affiliations:** 1 School of Biosciences, University of Birmingham, Edgbaston, Birmingham, B15 2TT UK; 2 School of Chemistry, University of Bristol, Cantock’s Close, Bristol, BS8 1TS UK; 3 College of Medicine, Kirkuk University, Kirkuk, Iraq; 4 Present Address: School of Chemistry, University of St Andrews, BMS Building, North Haugh, St Andrews, KY16 9ST UK; 5 Present Address: Dr M. Macioszek, DOCS International Poland, ul. Grojecka 5, 02-019 Warszawa, Poland; 6 Present Address: Ms M. di Martino, Dept Biology, University of York, Wentworth Way, York, YO10 5DD UK; 7 Present Address: Dr C. E. Miller, The BioHub Birmingham, Birmingham Research Park, 97 Vincent Drive, Edgbaston, Birmingham, B15 2SQ UK

## Abstract

The mupirocin *trans-*AT polyketide synthase pathway, provides a model system for manipulation of antibiotic biosynthesis. Its final phase involves removal of the tertiary hydroxyl group from pseudomonic acid B, PA-B, producing the fully active PA-A in a complex series of steps. To further clarify requirements for this conversion, we fed extracts containing PA-B to mutants of the producer strain singly deficient in each *mup* gene. This additionally identified *mupM* and *mupN* as required plus the sequence but not enzymic activity of *mupL* and ruled out need for other *mup* genes. A plasmid expressing *mupLMNOPVCFU* + *macpE* together with a derivative of the producer *P. fluorescens* strain NCIMB10586 lacking the *mup* cluster allowed conversion of PA-B to PA-A. MupN converts apo-mAcpE to holo-form while MupM is a mupirocin-resistant isoleucyl tRNA synthase, preventing self-poisoning. Surprisingly, the expression plasmid failed to allow the closely related *P. fluorescens* strain SBW25 to convert PA-B to PA-A.

## Introduction

Polyketide synthases (PKSs) are responsible for the synthesis of a huge diversity of secondary metabolites including antibiotics in clinical use, for example erythromycin^1–3^. Analysis of the genes encoding PKSs has revealed the way that this diversity has evolved via the combinatorial possibilities inherent in the backbone assembled by the PKS components and the recruitment of additional genes that are able to modify the backbone thus generating a range of novel structures^4^. Thus there is great potential for re-engineering PKS pathways to yield new molecules with novel biological activities^5,6^. In practice it has not been so easy to achieve this ambition. It is thought that a lack of structural understanding of these complex machines is what has confounded many attempts and so considerable effort has focused on understanding the way in which these complex structures are assembled^7^.

PKSs are broadly classified as type I or II depending on whether the individual enzyme activities are encoded as parts of multifunctional polypeptides (TI) or as individual polypeptides that assemble into multi-enzyme complexes (TII)^4^. The mupirocin system^8–10^ was one of the first discovered *trans*-acyl transferase (AT) TI PKSs in which the AT which loads the malonate extender unit onto Acyl Carrier Proteins (ACPs) of the biosynthetic factory is made as a separate polypeptide^11–13^. The mupirocin system consists of three polyketide synthases, MmpA, MmpD, MmpB, which create a 23 carbon backbone, and five auxiliary ACPs (mAcpABCDE) and further enzymes for regulating the gene cluster and tailoring the structure of the polyketide during and post synthesis, MupA through to MupZ^10^ (Fig. S1). Mupirocin has a number of chemical features whose biosynthetic machinery is not fully understood and which can potentially be added to a synthetic biology toolbox for the creation of novel pathways^10^.

**Figure 1:**
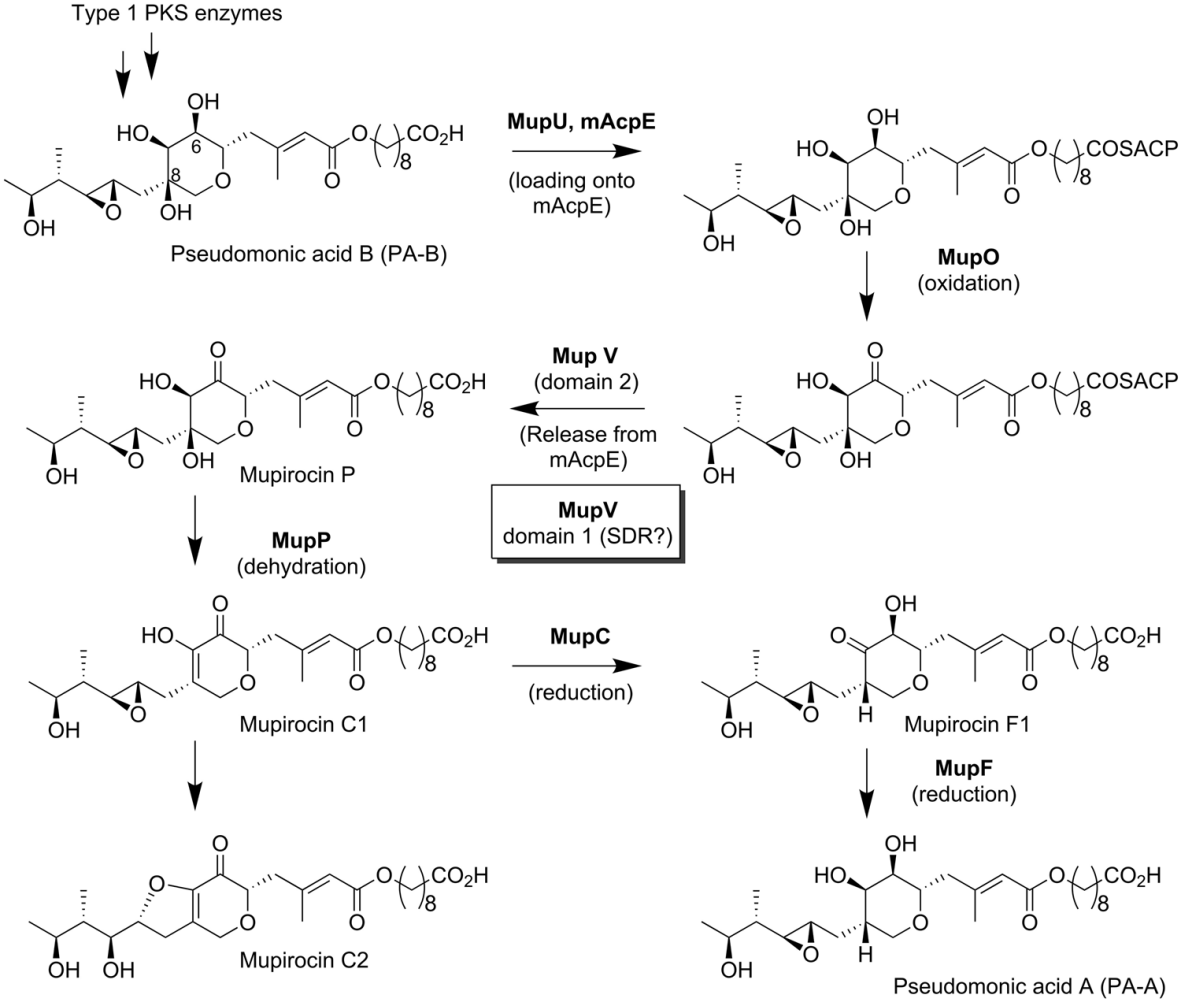
Proposed pathway for conversion of PA-B to PA-A.

One such feature is the tetrahydropyran ring that is essential for mupirocin activity, creating the structure that occupies the nucleotide binding site of the isoleucyl-tRNA synthetase active site^14^. MupW and T are necessary for tetrahydropyran ring formation but leave an hydroxyl group at C-8 of monic acid, characteristic of pseudomonic acid B^15–17^, as shown in Fig. 1. One of the surprises of our early mutational analysis of mupirocin biosynthesis was that PA-B might be an intermediate^15,18^, since mutants defective in *mupO*, *mupU*, *mupV* and *macpE* no longer produced PA-A but accumulated PA-B instead^15^, and this was confirmed by showing that mutants of genes implicated early in the pathway can convert PA-B to PA-A but that mutants of the later stages cannot^17,19^. Since all these mutants have the same phenotype their gene products were proposed to work together and based on predicted functions following BLAST analysis MupU (a predicted acyl-ACP synthase) was proposed to load PA-B onto mAcpE where it was oxidised by MupO (a P450 predicted oxidoreductase) before release by MupV, one domain of which is a predicted thioesterase. Further analysis of mutants of *mupC*^18^, *mupF*^17^ and *mupP*^19^, including double mutants with these PA-B-producer alleles, showed that MupC (a predicted enone reductase), MupF (a predicted ketoreductase) and MupP (a predicted dehydratase) act at a later stage. The structure of the intermediate Mupirocin P was characterised from a double mutant that cannot create the epoxide ring and this made the compound more stable^19^. Further isolation of other double mutants plus LC-MS and NMR elucidation of the structures of isolatable intermediates allowed prediction of the current scheme (Fig. 1). However, because this conversion has proven to be surprisingly complex and the current scheme for how this occurs may still not be the full story, this study aimed to define the full set of *mup* genes needed for conversion of PA-B to PA-A.

## Results

### Screening all mup cluster tailoring genes for PA-B to PA-A conversion

**Figure 2:**
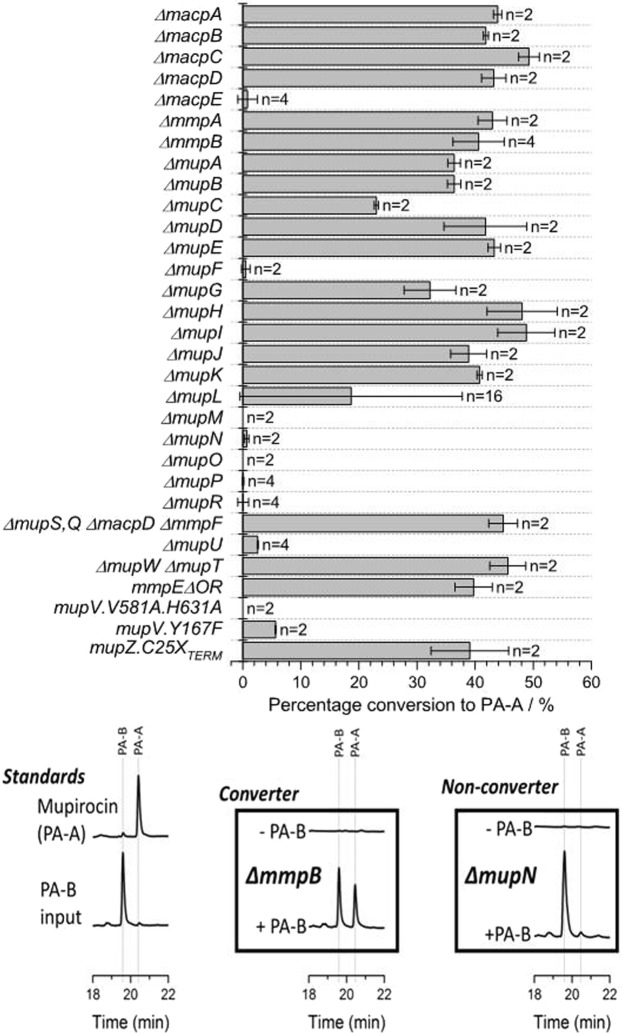
Results of HPLC screening of *mup* deletion mutants for PA-B to PA-A conversion. Most data are based on duplicate cultures (n = no. of replicates) except where results were variable, when larger numbers of replicates were performed. Error bars show the extreme values. For Δ*mupL*, values were either essentially zero or approximately 40%. Note that the tiny PA-A peak in the Δ*mupN* mutant is also present in the in-put PA-B profile.

To methodically identify all genes of the *mup* cluster that are required for PA-B to PA-A conversion we screened previously constructed mutant strains, each with a defect in one of the 35 mupirocin tailoring genes (Table S1) leading to loss of PA-A. For completeness we also included the strains that had previously been shown to accumulate PA-B. Cultures were grown as described in Methods with 0.5% (v/v) methanol or 0.5% PA-B extract in methanol, initially in duplicate, and supernatants analysed by HPLC. The amount of PA-A and PA-B present in each sample was determined by integration of HPLC peak areas. To determine the percentage of conversion of fed PA-B to PA-A, samples were first adjusted for their methanol controls, to account for any inherent production of either compound by the strains. This screening method proved successful, with a distinct difference in phenotype between converters and non-converters for the majority of genes (Fig. 2). Mutants of genes *macpE, mupF, mupO, mupP*, *mupU* and *mupV* were confirmed as deficient in conversion as expected based on published data^15,18,19^ and the cognate enzymes already appear in the current model while mutants of genes with characterised functions earlier in the pathway, such as *mmpA* or *mupW* and *mupT*, were capable of conversion as expected.

Mutants with defects in three further genes were consistently deficient in conversion: Δ*mupM*, Δ*mupN* and Δ*mupR*, although there may be a tiny amount of conversion in the *mupN* mutant. MupM is an isoleucyl tRNA synthetase that provides high level resistance to mupirocin; MupN is a phosphopantetheinyl transferase (PPTase); and MupR is the transcriptional activator required for mupirocin gene expression^20^. The lack of conversion by the Δ*mupM* strain prompted further investigation and we established that such strains stopped growing at lower density than WT, but remain viable, possibly the result of the stringent response due “starvation” for isoleucine. Thus the requirement for *mupM* is most likely to prevent this self-inhibition. The requirement for MupN in the conversion indicates for the first time that the standard PPTase of *P. fluorescens* cannot substitute for MupN and convert mAcpE to the holoenzyme, although the tiny amount of conversion observed could be due to very weak conversion of traces of mAcpE to holo-form. The ability of the Δ*mupI* mutant to convert was initially surprising since MupI, which produces homoserine lactone, is essential for activating all stages of mupirocin production. However, the presence of significant levels of homoserine lactone in the crude extract of PA-B was confirmed by showing that it could activate the *xylE* reporter gene integrated into *mupA* in a Δ*mupI xylE::mupA* strain which is defective in homoserine lactone production (Fig. S2)^21^.

Two further mutants demonstrated an intermediate phenotype, Δ*mupC* and Δ*mupL*. MupC is a predicted NADH:flavin oxidoreductase and possibly a dienoyl reductase while MupL is a putative alpha/beta hydrolase. The partial conversion of PA-B to PA-A by the *mupC* mutant agrees with the previous observation that such a mutant produces a lower titre of PA-A than WT, as well as intermediate metabolites mupirocin C1 and C2 (Fig. 1) which differ by the formation of a cyclic ether ring formed by the C7-OH attacking the C10-C11 epoxide ring^17^. It was deduced that the tautomerisation to produce mupirocin F1 can occur spontaneously but that MupC favours mupirocin F1 formation^17^. The ability of the Δ*mupL* mutant cultures to convert PA-B to PA-A was inconsistent, about half of all cultures (n = 16) converting and the others failing to. This variability was not due to a permanent change in the strain and was observed for all three independent Δ*mupL* mutant strains with the same, original in-frame deletion. This indicated that conversion could take place in the absence of a functional MupL protein and suggested that, since *mupL* is immediately upstream of *mupM*, the *mupL* deletion may remove sequences important for normal *mupM* transcription, possibly resulting in some cultures expressing *mupM* and others not doing so, and thus self-inhibiting. We concluded that *mupL* should be included upstream of *mupM* in the set of genes chosen for PA-B to PA-A conversion but that its requirement should be investigated more thoroughly at that stage.

### Both domains of MupV are required for PA-B to PA-A conversion

All the genes identified in the screen for PA-B to PA-A conversion described above are predicted to encode a single biochemical function apart from *mupV*. MupV was originally predicted to be an oxidoreductase^9^, but re-analysis of the amino acid sequence predicted two domains: the previously identified domain 1 predicted as a short chain dehydrogenase/reductase (SDR) (Blast E-value 1.46E–91) and a second domain predicted as an alpha-beta hydrolase or thioesterase (TE), (Blast E-value 9.12E–4). The hypothesis that MupV acts as a thioesterase^19^ provides a mechanism for release of the polyketide from mAcpE but does not explain the presence of two domains. Therefore, point mutations to conserved residues were designed to test whether the functions of both are required for conversion of PA-B to PA-A. For domain 1 (SDR) the Tyr176 in the active site NAD-binding Rossman fold YxxxK motif ^22^ was changed to Phe, minimising the chance of structural changes to the enzyme. There are no predicted well-characterised active site motifs in Domain 2 (TE) but alignment of 55 related sequences revealed a strongly conserved His which was mutated to Ala (Fig. S3). Overlap extension PCR was used to generate mutagenic segments that were inserted into the suicide vector pJC70 and the mutations transferred into the chromosome of NCIMB10586 using standard procedures. A second mutation in domain 2, V581A, occurred by chance in the mutagenic suicide plasmid and all clones identified with H631A also had the V581A mutation. Because some clones had the V581A mutation on its own we decided to see whether this single mutation alone had any effect on MupV activity before starting again to make the mutagenic suicide plasmid. The three *mupV* mutant strains were assayed for active mupirocin production with *Bacillus subtilis* as the indicator. Both the Y167F and H631A (with V581A) mutations decreased the zones of inhibition compared with WT, whereas the V581A mutation showed no effect (Fig. S4), consistent with an I-Tasser 3D model of MupV which predicts that this residue is situated well away from the active site. HPLC analysis (Fig. S5) followed by LC-MS and ^1^H-NMR (Fig. S6) demonstrated that both domains are required for conversion of PA-B to PA-A. This suggests that either both domains are involved in the proposed release from MacpE, or that MupV has an additional role in PA-B to PA-A conversion.

### Conversion of PA-B to PA-A achieved by selected mup genes in an expression plasmid

**Figure 3:**
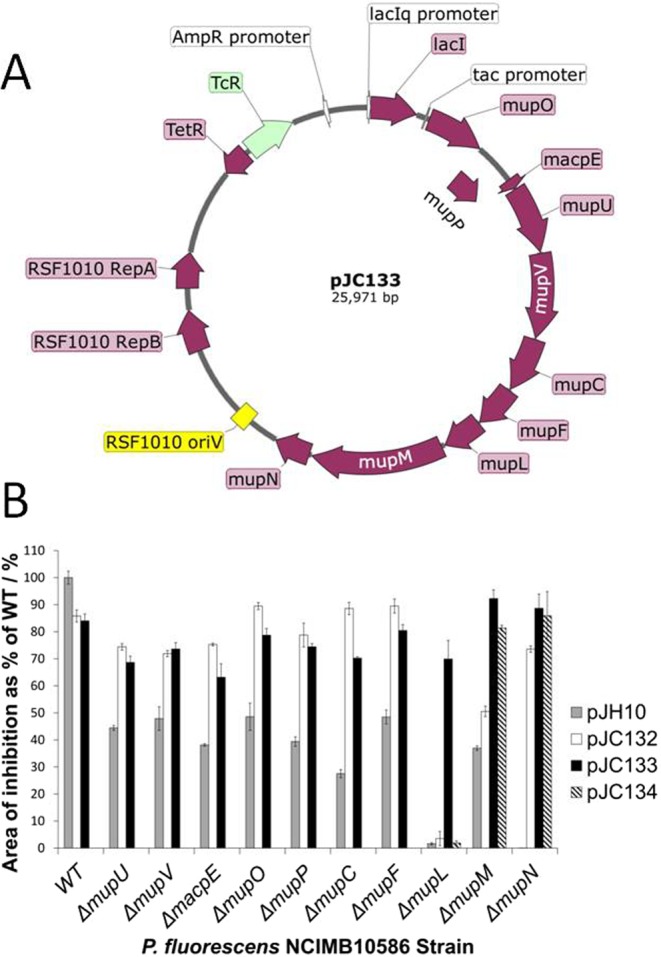
Expression plasmid with all *mup* genes needed for conversion of PA-B to PA-A. (**A**) Plasmid map. (**B**) Functional bioassays for each gene in the constructed plasmids (see Table 1) using *B. subtilis*; the best performing IPTG induction condition is shown, which was 0.5 mM IPTG for Δ*mupM* [pJC132] and Δ*mupN* [pJC132] and 0 mM IPTG for all other samples; n = 3, error bars are standard deviation. For Δ*mupN* the strain with pJH10 gave so little activity the column is not visible.

To check whether the genes identified by the screening described above are sufficient for PA-B to PA-A conversion, we constructed a broad host range expression plasmid with all these genes spliced together as an operon downstream of the *tac* promoter (pJC133; Fig. 3A and Table 1). Plasmid pJC133 was constructed carrying genes *mupO, P, macpE, mupU, V, C, F, L, M and N*. To determine the need for the *mupL* sequence, a variant without *mupL* was constructed, pJC132. The *mupL, M, N* block was added at the downstream end because if the natural complete gene sequence from *mupL* to *mupP* had been included as the start of the operon we worried that there could be a problem due to an inverted repeat downstream of *mupN* that might be a transcriptional terminator.

**Table 1:** Summary of expression plasmids in this study.

Plasmid	Mupirocin genes carried	PA-B to PA-A?
pJH10	none	✗
pJC132	*mupO*, *P*, *macpE*, *mupU*, *V*, *C*, *F*, *M*, *N*	✗
pJC133	*mupO*, *P*, *macpE*, *mupU*, *V*, *C*, *F*, ***L***, *M*, *N*	✓
pJC134	*mupO*, *P*, *macpE*, *mupU*, *V*, *C*, *F*, ***L***^**−**^, *M*, *N* (*mupL* inactivated by point mutation)	✓

The functionality of each gene in each plasmid was checked by complementation and plate bioassay for antibiotic production performed with and without IPTG (0.5 mM; Fig. 3B). The ability to complement PA-A production in *mupL*, *mupM* and *mupN* in-frame deletion mutants was also checked by HPLC (Fig. S7). Without IPTG pJC133 could complement all three mutants, while pJC132 could only complement the *mupN* mutant despite the presence of what should be the complete *mupM* sequence including its ribosome binding site, suggesting a defect in expression of *mupM*. With IPTG a *mupM* mutant could also be at least partially complemented. This suggests that the *mupL* sequence is necessary to achieve sufficient expression of *mupM* for complementation but that *mupN* is either not dependent in the same way (perhaps there is an additional promoter within *mupM* for *mupN*) or that lower levels of *mupN* expression are sufficient for complementation. An inverted repeat downstream of *mupM* in the gap before *mupN* could potentially influence the *mupM* mRNA separately from the *mupN* mRNA but there is no obvious promoter-like sequence that could serve *mupN* but not *mupM* and detailed analysis is beyond the scope of this paper. A point mutation (H256A) that should inactivate the enzymic activity of MupL was introduced into pJC133 (giving pJC134) to test the prediction that this region is only needed to allow *mupM* expression. This mutation destroyed the ability to complement the *mupL* deletion mutant but the *mupM* functionality was maintained (Fig. 3B) in line with our prediction that *mupL* may contain a promoter for *mupM*. The 3′ end of the *mupL* gene contains a number of possible promoter-like sequences and inverted repeats that may be relevant but experimental evidence to distinguish the various explanations is not yet available. However, none of these potential promoters contains the sequences that we predict are the *mupR* binding site, suggesting that basal *mupM* expression is constitutive rather than quorum-regulated. Higher level of *mupM* expression than for the other *mup* genes may be necessary to ensure that the bacterium can continue to grow once mupirocin production is initiated.

**Figure 4:**
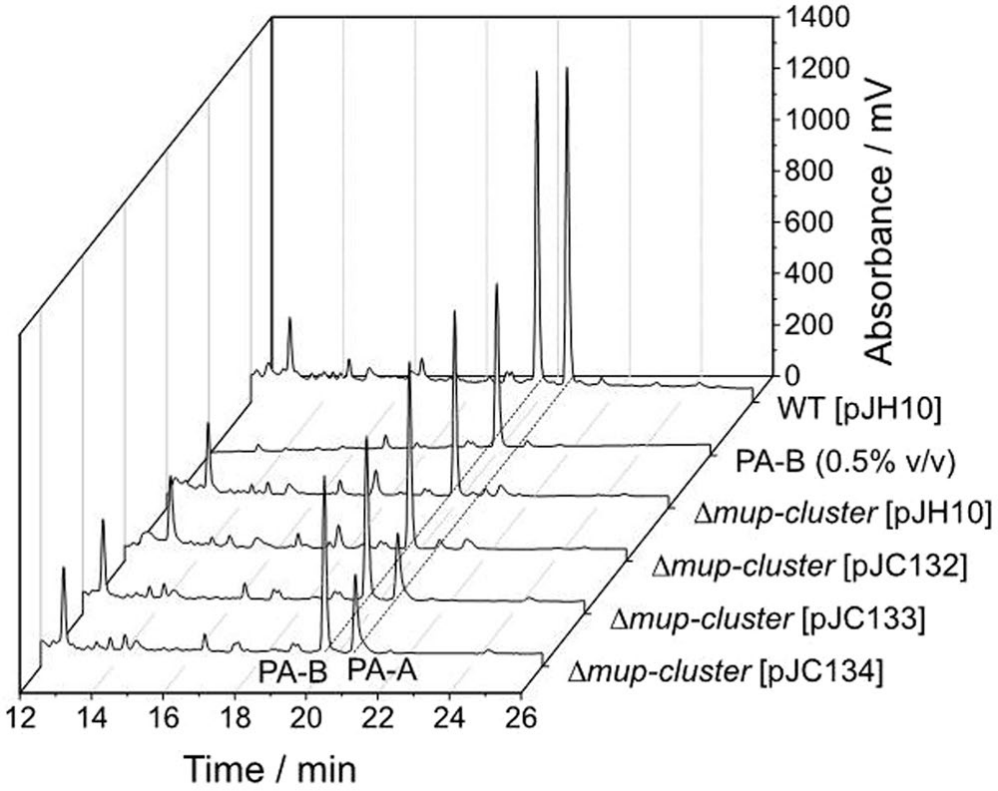
HPLC analysis of extracts from cultures grown without added IPTG showing multi-gene expression plasmids pJC133 and pJC132 encode all functions necessary to restore conversion of exogenous PA-B to PA-A in 10586 Δ*mup-cluster*. No conversion was observed with plasmid pJC132, which lacks the *mupL* gene.

Finally, pJC132, pJC133 and pJC134 were all introduced into a NCIMB10586 deletion derivative that had lost all of the *mup* cluster biosynthetic genes (as opposed to regulatory genes) from the first gene, *mupZ*, to *mupW*, the last gene before the *mupRXI* regulatory cluster. The ability to convert PA-B to PA-A was tested in the presence (0.5 mM) and absence of IPTG. The presence of both pJC133 and pJC134 but not pJC132 allowed conversion in the absence of IPTG but none of the plasmids were able to do that in the presence of IPTG (Fig. 4) indicating that low level expression from the leaky *tac* promoter is sufficient for the majority of the genes is sufficient for function. These plasmids were also introduced into the closely related strain *P. fluorescens* SBW25^23^ but this host was found not to allow any of the plasmids to catalyse conversion of PA-B to PA-A.

## Discussion

The work described in this paper has systematically demonstrated for the first time which genes from the *mup* cluster are necessary for PA-B to PA-A conversion during mupirocin biosynthesis and which genes are not. Gratifyingly, it turns out that we had already identified all the enzymes directly involved in the PA-B to PA-A conversion encoded by genes of the *mup* cluster, so the proposed pathway in Fig. 1 still functions as a working model. This does not mean that no questions remain.

Our previous studies with *mupN*, which encodes an Sfp-like PPTase^24^, had shown only that it is needed to activate at least some of the ACPs encoded in the *mup* cluster^18,25^. Shields *et al*.^25^ showed that, at least in *E. coli*, some *mup* ACPs are not totally dependent on MupN: when the *macpA* gene was expressed in *E. coli* in the absence and presence of cloned *mupN*, mass-spectrometry detected mAcpA as holo-ACP even without MupN present. In the Shields *et al*.^25^ study, mAcpE had proven impossible to produce in soluble form and so the requirements for its conversion to holoenzyme could not be determined. In the current study the only ACP required for the PA-B to PA-A conversion is mAcpE and so the simplest explanation for the additional requirement for *mupN* is that it is essential for conversion of mAcpE to its active holo- (phosphopantetheinylated) form. Understanding of the code that determines the specificity of different PPTases on ACPs is not yet complete enough to spell out the basis of this requirement^24^.

Our bioinformatic and mutational analysis shows that MupV (662 aa) consists of two distinct domains which are both necessary for the transformation. Domain 1 (approximately amino acids 1–320) is predicted to belong to the short-chain dehydrogenase/reductase (SDR) superfamily while domain 2 is predicted to be an α/β hydrolase. Although there is not yet any direct evidence for the role of MupV, the absence of MupV and mAcpE homologues encoded by the biosynthetic cluster for the related polyketide antibiotic thiomarinol^10^ and an extra in *cis* module in TmpB^26^ suggest that removal of the 8-OH may occur on the ACP of this second TmpB module so that mAcpE and MupV are not needed. On this model the domain 2 seems most likely to be a thioesterase that is needed to release products from mAcpE^27^. The potential role of domain 1 is more enigmatic but since the SDR family includes enzymes that do neither oxidation nor reduction^22^, a possibility is that it works with MupU in loading mAcpE. Thus, as proposed for TmlU in thiomarinol biosynthesis^28^, rather than transferring PA-B direct to mAcpE, MupU may activate PA-B by transfer to coenzyme A and MupV domain 1 may transfer it to mAcpE. Such a dual role for a single bi-domain protein in loading/off-loading ACPs might make a lot of sense in other contexts and interestingly a Blast search shows that there are numerous distantly related proteins that also have both of these functions paired in a single dual-domain protein. Another possibility is that mupirocin P arises from the dual action of MupO and MupV rather than just MupO. So far this has proven intractable as *mupV* knockouts simply yield PA-B (Fig. S6) and shed no light on the “true” MupO product.

It is also intriguing that pJC133 encoding the complete set of genes could not convert PA-B to PA-A in *P. fluorescens* SBW25 and this would be consistent with the identification of transposon insertion mutants of NCIMB10586 defective in mupirocin production and mapping away from the cluster of insertions that defined the region encoding the *mup* cluster we have studied for many years^29^. The problem with *P. fluorescens* SBW25 could be simply that PA-B cannot enter or PA-A cannot exit efficiently. Alternatively, one or more components encoded elsewhere on the genome in NCIMB10586 may be necessary for the conversion. The finding that MupM is essential for the conversion and that the *mupL* sequence is important in giving a consistent ability to convert PA-B to PA-A, can underpin future work to define additional components if none of the mutants described by Whatling *et al*.^29^ identify the locus required. A new transposon insertion library could be screened for strains that are not adversely affected by feeding PA-B: mutants that are not able to make the inhibitory PA-A will be enriched. Thus, the analysis we have described lays the basis for working out, not just what the Mup proteins involved are doing but how the pathway fits into the broader context of its bacterial host.

## Methods

### Bacterial strains, plasmids and growth conditions

Bacterial strains are listed in Supplementary Table S1. Plasmids used and constructed in this study are listed in Supplementary Table S2. Oligonucleotides are listed in Supplementary Table S3. Routine methods were as described previously^30^. Bacteria were routinely cultured at either 37 °C (*E. coli* and *B. subtilis*) or 30 °C (*P. fluorescens*) in lysogeny broth (LB) with shaking at 200 rpm or on Lagar (LB + 1.5% w/v agar). *P. fluorescens* was grown on standard M9 minimal medium agar with glucose to counter-select against *E. coli*. Prior to assay of secondary metabolite production by HPLC, *P. fluorescens* was cultured in Secondary Stage Medium (SSM). Antibiotics used were ampicillin (100 µg ml^−1^) and kanamycin (50 µg ml^−1^).

To prepare SSM, a mixture of 25 g soya flour, 2.5 g spray dried corn liquor, 5 g (NH_4_)_2_SO_4_, 0.5 g MgSO_4_.7H_2_O, 1 g Na_2_HPO_4_, 1.5 g K_2_HPO_4_, 1 g KCl, and 6.25 g CaCO_3_ was generated. The pH of the mixture was adjusted to 7.5, made up to 1 L dH_2_O and autoclaved. Before use 40% (w/v) glucose was added to the SSM to give a final concentration of 4%.

### Conjugative transfer of plasmids to *P. fluorescens*

Plasmids carrying *oriT* were first introduced into *E. coli* S17-1^31^ by transformation, which has the RP4 *tra* regions integrated into the chromosome, enabling conjugative transfer of plasmids with *oriT*. Overnight (16 hour) cultures of donor S17-1 carrying the relevant plasmid were grown in LB with antibiotic selection for the plasmid. Simultaneously, 16 hr cultures of the recipient *P. fluorescens* strains were grown in LB. To allow conjugation to occur, 10 µl of both donor and recipient cultures were spotted on top of each other on L-agar and then incubated at 30 °C for 12–20 hours. The mixed cultures were streaked to single colonies on L-agar with ampicillin to select for *P. fluorescens*, and the antibiotic selective for the plasmid. Transconjugants were again streaked onto selective plates to minimise the risk of *E. coli* donor contamination.

### Mutagenesis of *P. fluorescens*

Suicide plasmids were mobilised to *P. fluorescens* by conjugation from *E. coli* S17-1. Transconjugants were selected with ampicillin or on M9 minimal medium (for *P. fluorescens*) and kanamycin (for pAKE604- or pJC70-derived plasmids). These plasmids have the pMB1 replicon, which does not function in *P. fluorescens*. Therefore, kanamycin selects for plasmids that have integrated into the *Pseudomonas* chromosome by homologous recombination. Transconjugant colonies were re-streaked to ensure they were free of the donor *E. coli* strain and then grown in LB without selection for 16 hours to allow a second recombination event to occur. The vectors include *sacB*, which encodes levansucrase from *Bacillus subtilis*, which confers a lethal periplasmic sucrose polymerisation in Gram negative bacteria^32^. Serial dilutions of these cultures were plated onto L-agar with 5% (w/v) sucrose, which selects for bacteria that have lost *sacB*. Sucrose resistant colonies were then patched onto L-agar with and without kanamycin, to check for plasmid loss. Since the integration and excision events have an equal chance of occurring in either homology arm flanking the mutation, about 50% of the excisants should contain the mutation. Mutant strains were detected by PCR, restriction digest and sequencing. Strains with multiple genes knocked out were either made by a single deletion if the genes were adjacent (eg Δ*mupQ*, *mupS*, *macpD*, *mmpF*) or by sequencial knockouts (eg Δ*mupW*, Δ*mupT*).

### Extraction of PA-B

Ethyl acetate extraction was used to concentrate PA-B for use in the feeding experiments. Plasmid pJH2 (IncQ vector with *mupR* under *tac* promoter control) was mobilised to the PA-B-producing strain NCIMB10586 Δ*mupU*, as expression of transcription factor MupR increases secondary metabolite yield^18^. Seed cultures were grown overnight at 30 °C in LB. SSM cultures (200 ml) in 2 L conical flasks, were inoculated with 2 ml of saturated seed culture, and incubated at 22 °C for 40–44 hours.

Cultures were aliquoted into 50 ml Falcon conical centrifuge tubes and centrifuged at 3000 × g for 10 minutes at room temperature. The supernatants were collected and pH adjusted to 4.5, before being split into aliquots in 50 ml Falcon tubes, approximately half full (25 ml). To each tube was added approximately 25 ml ethyl acetate (EtOAc), mixed thoroughly by inversion and vortexing approximately ten times over half an hour. Where emulsions were formed, these were resolved to layers by centrifugation (3000 × g, 5 minutes). The clear top layer was collected using a glass pipette. Each tube was refilled with EtOAc, and the process repeated, again collecting the top layer. The collected solvent was evaporated on a rotary evaporator until <500 µl oily yellow liquid remained. Methanol (2 to 4 ml) was added and any precipitated solids dissolved by pipetting. The purity and concentration of the extract was determined by HPLC analysis.

### High performance liquid chromatography

To prepare samples for HPLC analysis, *P. fluorescens* 5 ml LB seed cultures, with suitable antibiotics, were inoculated in triplicate from single colonies on solid medium and incubated for 16 hours at 30 °C. Seed cultures (200 µl) were used to inoculate 5 ml SSM in 50 ml conical flasks, which were incubated at 22 °C 200 rpm for 44 hours. Samples were centrifuged at 13,000 g and supernatants collected. The supernatants were filtered through 0.2 µm millipore filters prior to injection.

HPLC analysis was performed on a Gilson system using a reverse phase C18 column. Mobile phase was a water:acetonitrile gradient, starting from 95:5 (t = 0 minutes) to 30:70 (t = 30 minutes). Both the HPLC grade water and acetronitrile were made up with 0.01% formic acid. Compounds were detected by UV absorption at 233 nm.

### PA-B feeding experiments

To screen bacterial strains for their ability to convert PA-B to PA-A SSM cultures (5 ml) were supplemented with PA-B extract (at 0.5 to 1.0% (v/v) to give a concentration of approximately 150 µM) or an equivalent amount of methanol as a negative control, and incubated at 22 °C 200 rpm for 44 hours before HPLC analysis. Where necessary IPTG was also added at 0.5 mM to induce expression from the *tac* promoter.

### Plate bioassay

Plate bioassays were used to determine the antibiotic activity of test *P. fluorescens* strains against the mupirocin-sensitive strain *B. subtilis* 1604. Cultures of *P. fluorescens* were grown for 16 hours with suitable antibiotics in LB; 10 µl of these were spotted onto 20 ml measured L-agar plates, and incubated for 16 hours at 20 °C. *B. subtilis* overnight culture (8 ml) was mixed with 1 ml 5% triphenyl tetrazolium chloride (TTC) in 200 ml L-agar. Each plate was overlaid with 15 ml of the *B. subtilis-*containing mixture. Plates were incubated for 16 hours at 37 °C, and the diameters of the zones of inhibition were measured.

### Chromogenic xylE reporter assay

NCIMB10586 Δ*mupI mupA::xylE* has the reporter gene *xylE* inserted downstream of the putative *mupA* promoter^21^ and is defective in the cognate homoserine lactone (HSL) quorum sensing signal molecule. The level of *xylE* expression was used to detect exogenous HSL. To perform the assays, overnight LB cultures of the reporter and relevant test strains were streaked onto SSM agar plates, which were incubated for 22 hours at 20 °C. Plates were then mist-sprayed with 1% (w/v) catechol in SDW, and the colour change was observed after 5 minutes. Colourless catechol is converted to yellow 2-hydroxymuconic semialdehyde, catalysed by XylE^33^, allowing for detection of transcription from the *mupA* promoter.

### Quantification and statistical analysis

HPLC data were exported from the Gilson Unipoint software that operates the machinery to OriginPro2016. Peak areas were calculated in OriginPro2016 using the inbuilt Peak Analyzer tool with the baseline mode set to asymmetric least squares smoothing.

To determine the percentage of conversion of fed PA-B to PA-A in the Fig. 2 dataset, samples were first adjusted for their methanol controls, to account for any inherent production of either compound by the strains. Percentage conversion was calculated as the amount of PA-A produced divided by the total pseudomonic acids produced (PA-A and PA-B).

## Supplementary information

Supplementary Information
