# Supplementary material for: Defining the genes for the final steps in biosynthesis of the complex polyketide antibiotic mupirocin by Pseudomonas fluorescens NCIMB10586
Source: Sci Rep. 2019 Feb 7;9:1542. doi: 10.1038/s41598-018-38038-9 (PMC6367315; doi:10.1038/s41598-018-38038-9)

Defining the genes for the final steps in biosynthesis of the complex polyketide antibiotic mupirocin by *Pseudomonas fluorescens* NCIMB10586

Jack A. Connolly [a], Amber Wilson[a], Malgorzata Macioszek[a][c], Zhongshu Song[b], Luoyi Wang[b],], Hadi H. Mohammad[a], Mukul Yadav[a], Maura di Martino[a][d], Claire E. Miller[a], Jo Hothersall[a], Anthony S. Haines[a], Elton R. Stephens[a], Matthew P. Crump[b], Christine L. Willis[b], Thomas J. Simpson[b], Peter J. Winn[a] and Christopher M. Thomas*[a].

Supplementary Methods

Table S1. Bacterial strains used and constructed in this work.

Bacterial Strains	Source/Reference	Notes
Escherichia coli DH5α	Thermofisher	Cat#18263-012
E. coli S17-1 (RP4 *tra*)	Ref. 31	N/A
E. coli ER2925 (Dam$^-$ and Dcm$^-$)	New England Biolabs	Cat#E4109
Bacillus subtilis 1604/168	ATCC23857	Mupirocin sensitive
Pseudomonas fluorescens SBW25	Ref. 33	WT Non-producer
Pseudomonas fluorescens NCIMB10586	NCIMB ref. 8	WT mupirocin producer
P. fluorescens NCIMB10586 Δ*mupI xylE::mupA*	Ref. 21	Reporter *xylE* gene in *mupA*
P. fluorescens NCIMB10586 Δ*mupZ-mupI*	Claire Miller, this work	Lacks the *mup* cluster
P. fluorescens NCIMB10586 Δ*macpA*	Ref. 18	No production
P. fluorescens NCIMB10586 Δ*macpB*	Ref. 18	No production
P. fluorescens NCIMB10586 Δ*macpC*	Ref. 18	Produced Mupirocin H and mupiric acid
P. fluorescens NCIMB10586 Δ*macpD*	Ref. 18	No production
P. fluorescens NCIMB10586 Δ*macpE*	Ref. 18	Produces PA-B
P. fluorescens NCIMB10586 Δ*mmpA*	Ref. 17	Produces mupircin acid
P. fluorescens NCIMB10586 Δ*mmpB*	Jo Hothersall, this work	Produces mupircin acid
P. fluorescens NCIMB10586 *mmpE*ΔOR	Ref. 17	Produces PA-C
P. fluorescens NCIMB10586 Δ*mupA*	Jo Hothersall, this work	Produces mupiric acid
P. fluorescens NCIMB10586 Δ*mupB*	Jo Hothersall, this work	No PA-A production
P. fluorescens NCIMB10586 Δ*mupC*	Ref. 18	Produces Mupirocin C
P. fluorescens NCIMB10586 Δ*mupD*	Ref. 18	No PA-A production
P. fluorescens NCIMB10586 Δ*mupE*	Ref. 18	No PA-A production
P. fluorescens NCIMB10586 Δ*mupF*	Ref. 18	Produces Mupirocin F
P. fluorescens NCIMB10586 Δ*mupG*	Ref. 18	Produces Muprocin H
P. fluorescens NCIMB10586 Δ*mupH*	Ref. 18	Produces Muprocin H
P. fluorescens NCIMB10586 Δ*mupI*	Ref. 21	No production
P. fluorescens NCIMB10586 Δ*mupJ*	Ref. 18	Produces Muprocin H
P. fluorescens NCIMB10586 Δ*mupK*	Ref. 18	Produces Muprocin H
P. fluorescens NCIMB10586 Δ*mupL*	Ref. 18	N o production
P. fluorescens NCIMB10586 Δ*mupM*	Ref. 18	No production
P. fluorescens NCIMB10586 Δ*mupN*	Ref. 18	No production
P. fluorescens NCIMB10586 Δ*mupO*	Ref. 15	Produces PA-B
P. fluorescens NCIMB10586 Δ*mupP*	Ref. 18	Produces Mupirocin P
P. fluorescens NCIMB10586 Δ*mupR*	Ref. 21	No production
P. fluorescens NCIMB10586 Δ*mupQ,* Δ*mupS,* Δ*macpD,* Δ*mmpF*	Mukul Yadav, this work	Produces Mupirocin H and mupiric acid
P. fluorescens NCIMB10586 Δ*mupU*	Ref. 15	Produces PA-B

P. fluorescens NCIMB10586 Δ*mupW*	Ref. 15	Produces Mupirocin W
P. fluorescens NCIMB10586 Δ*mupW,* Δ*mupT*	Jo Hothersall, this work	Produces Mupirocin W
P. fluorescens NCIMB10586 Δ*mupX*	Ref. 15	Reduced PA-A production
P. fluorescens NCIMB10586 *mupV* Y167F	Jack Connolly, this work	Produces PA-B
P. fluorescens NCIMB10586 *mupV* V581A H631A	Jack Connolly, this work	Produces PA-B
P. fluorescens NCIMB10586 *mupZ* C25X (TERM)	Hadi Mohammed, this work	No PA-A production

Table S2. Plasmids used and constructed in this work.

Plasmid	Source/Reference	Notes
pAKE604	Ref. 21	Km^R Ap^R pMB1 replicon, *oriT*$_{RK2}$
pJC70	Jack Connolly, this work	Km^R Ap^S derivative of pAKE604
pGEM-T Easy	Promega Cat#A1360	Ap^R pMB1 replicon linear T-tailed vector
pJH2	Ref. 18	pJH10 expressing *mupR*
pJH10	Ref. 9	Tc^R IncQ vector with *tac*p *lacI*^{q} expression
pAW1	This work	pJC70 derivative for *mupV* domain 1 Y167F mutagenesis
pJC102	This work	pJC70 derivative for *mupV* domain 2 V581A, H631A mutagenesis
pHHMZC25	This work	pAKE604 derivative for *mupZ* mutagenesis
pMY21	This work	pAKE604 derivative to delete *mupQ mupS macpD mmpF*
pSCΔT	Ref. 15	pAKE604 derivative to delete *mupT*
pMMG1	This work	pGEM-T derivative with *mupO*
pMMG2	This work	pGEM-T derivative with *macpE*
pMMG12	This work	pGEM-T derivative with *mupO, macpE*
pMMG34	This work	pGEM-T derivative with *mupU, mupV*
pMMG1234	This work	pGEM-T derivative with *mupO, macpE, mupU, mupV*
pMMG5	This work	pGEM-T derivative with *mupC*
pMMG6	This work	pGEM-T derivative with *mupF*
pMMG56	This work	pGEM-T derivative with *mupC, mupF*
pMMG66	This work	pGEM-T derivative with *mupO, macpE, mupU, mupV, mupC, mupF*
pMMH6	This work	pJH10 derivative expressing *mupO, macpE, mupU, mupV, mupC, mupF*
pJC124	This work	pJH10 derivative expressing *mupO, mupP, macpE, mupU, mupV, mupC, mupF*
pJC132	This work	pJH10 derivative expressing *mupO, mupP, macpE, mupU, mupV, mupC, mupF, mupM, mupN*
pJC133	This work	pJH10 derivative expressing *mupO, mupP, macpE, mupU, mupV, mupC, mupF, mupL, mupM, mupN*
pJC134	This work	pJH10 derivative expressing *mupO, mupP, macpE, mupU, mupV, mupC, mupF, mupL*-H256A, *mupM, mupN*

Table S3. Oligonucleotides used in this work.

Oligonucleotides: Name and sequence	Source	Purpose
AW1 AGACAATTGAGCAGGCCCGTCATTTAC	This work	*mupV* mutagenesis, pAW1
AW2 TGGTTTCTTCGAAATAGTTATTGAATTCAGTGC	This work	*mupV* mutagenesis, pAW1
AW3 CAATAACTATTTCGAAGAAACCAAATGTGCG	This work	*mupV* mutagenesis, pAW1
AW4 AGAGGATCCGTTGTGGATGTAGGGGTTGTAAAAC	This work	*mupV* mutagenesis, pAW1
JC1 GGAATCGTCGACAAAGCACATCTGATCGGCT	This work	*mupV* mutagenesis, pJC102
JC2 CAAACCAAATGCATCCATGCCAGCGATTTC	This work	*mupV* mutagenesis, pJC102
JC3 GGCATGGATGCATTTGGTTTGTATAACCACCTGG	This work	*mupV* mutagenesis, pJC102
JC4 GCACTGGAATTCTGACGACAACTGTTGTAGTAGGC	This work	*mupV* mutagenesis, pJC102
QDA1F GGTGAATTCGAAGATCTGGAGGACGCCGT	This work	Deletion of *mupS*,*Q macpD*, *mmpF*
QDA1R TGGTCTAGAGTCGCTGATCCAATTACGTTCCTC	This work	Deletion of *mupS*,*Q macpD*, *mmpF*
QDA2F GGTTCTAGAGACGTTGTGCAGGCCGGAATC	This work	Deletion of *mupS*,*Q macpD*, *mmpF*
QDA2R TGGGGATCCGCTCACGTTGGGCGAACCAC	This work	Deletion of *mupS*,*Q macpD*, *mmpF*
HHMZ1F TCAGGATCCGAGAGCATCTGCCTTACAAGC	This work	*mupZ* mutagenesis, pHHMZC25
HHMZ1R GATAAACACGGCTCAATACGC	This work	*mupZ* mutagenesis, pHHMZC25
HHMZ2F GCGTATTGAGCCGTGTTTATC	This work	*mupZ* mutagenesis, pHHMZC25
HHMZ2R GAGTCTAGAGAGTGACGCCGCCATTAC	This work	*mupZ* mutagenesis, pHHMZC25
FΔbla TCCGAGCTCCCAGTTCGATGTAACCCACTC	This work	Deletion of *bla* from pAKE604
RΔbla GAAGAGCTCAGGCAACTATGGATGAACG	This work	Deletion of *bla* from pAKE604
FmupO GGTACCGGAGAGCAACGATGACATCGTGGGAAAGAGA	This work	*mupO* amplification
RmupO ACCGGTTACCGGGCGATTCCGTGG	This work	*mupO* amplification
FmacpE ACCGGTCACAGGAGGCGTAGATGC	This work	*macpE* amplification
RmacpE ACTAGTTCTAGACTTAAGTCACTGCTCACGTTGGGC	This work	*macpE* amplification
FmupUV CTTAAGGGCAAGGGAATGTGAGATG	This work	*mupUV* amplification
RmupUV ACTAGTTCTAGAAAGCTTTTATGCATCCGCCTGGGA	This work	*mupUV* amplification
FmupC AAGCTTCGGAACGAGGCCGCCACC	This work	*mupC* amplification
RmupC CCTAGGGGATCCTCAGCGCTTATAGGTTGAGCA	This work	*mupC* amplification
FmupF CCTAGGCCCAAGGATCGCTACCGA	This work	*mupF* amplification
RmupF ACTAGTTCTAGATCAGGGTGCTGTCGGGGA	This work	*mupF* amplification
FmupOP GAACGGTACCTACTAGTGGGAGAGCAACGATGACATC	This work	*mupOP* amplification
RmupOP GCCTCCTGTGGTGTGCTCCTGTTGCTGTTTG	This work	*mupOP* amplification
FmacpE2 ACAGGAGCACACCACAGGAGGCGTAGATGC	This work	*macpE* amplification
RmacpE2 AGACTTAAGTCACTGCTCACGTTGGGC	This work	*macpE* amplification
FmupLMN CCACTCTAGACAGTGAAGAGAGGGACACACAGC	This work	*mupLMN* amplification
Rmup(L)MN GGTCTCAGGCCGCGATCCAAACCTATAAGCACTG	This work	*mupLMN* and *mupMN* amplification
FmupMN TAGCTCTAGACTGACAGGTGTGACTGATGAGTACG	This work	*mupMN* amplification
FmupL-H256A GATTGGGGGGCCTACACGCTTTTCTCTGATACACAAGAG	This work	Mutation of *mupL*
RmupL-H256A AGCGTGTAGGCCCCCCAATCGTCCATGATG	This work	Mutation of *mupL*

Construction of plasmids created during this work

Construction of mutagenesis plasmids

All plasmid constructs reported in this paper were checked by suitable restriction digests and Sanger sequencing of inserted DNA.

pAW1 – *mupV* domain 1 Y167F

Primer pairs AW1 plus AW2 and AW3 plus AW4 were used to amplify 555 bp and 502 bp segments of the NCIMB10586 chromosome, respectively. Primers AW2 and AW3 were designed to generate a 23 bp overlap, which included the *mupV* Y167F mutation (DNA codon TAC to TTC). The products were spliced by overlap PCR using primers AW1 and AW4, and cloned using MfeI/BamHI digestion and ligation with pAKE604 digested with *Eco*RI/*Bam*HI to yield mutagenesis vector pAW1.

pHHMZC25 – mupZ C25X (TERM)

Primers HHMZ1F plus HHMZ1R and HHMZ2F plus HHMZ2R were used to amplify 546 bp and 582 bp fragments from the NCIMB10586 chromosome. Primers HHMZ1R and HHMZ2F were designed to generate a 21 bp overlap which contained the base to be mutated from C to A, generating a TGA stop codon. The products were spliced by overlap PCR using primers HHMZ1F and HHMZ2R, and cloned using BamHI/XbaI digestion and ligation with pAKE604 digested with *Eco*RI/*Bam*HI to yield mutagenesis vector pHHMXC25.

pJC70 – derivative of suicide plasmid pAKE604 lacking the *bla* gene that confers Ap^R

To allow easy selection of transconjugants after transfer of suicide mutagenesis plasmids from *E. coli* to *P. fluorescens* (which is naturally Ap^R) the *bla* gene was deleted. Primers FΔbla and RΔbla were used to amplify the 6574 bp segment of pAKE604 that excludes the *bla* gene, recircularised by ligation after cutting with SacI and introduced into *E. coli* DH5 selecting Km^R. A correct clone not conferring Ap^R was designated pJC70.

pJC102 – *mupV* domain 2 V581A, H631A

Primer pairs JC1 plus JC2 and JC3 plus JC4 were used to amplify 546 bp and 500 bp segments of the NCIMB10586 chromosome, respectively. Primers JC2 and JC3 were designed to generate a 21 bp overlap, which included the *mupV* H631A mutation (DNA codon CAT to GCA). The products were spliced by overlap PCR using primers JC1 and JC4, and then A-tailed and ligated into pGEM-T Easy (Promega). Sequencing revealed a secondary, unintended point mutation (V581A), which was deemed acceptable as it was also in domain 2 of *mupV*. The DNA fragment was cloned using *Sal*I/*Eco*RI digestion and ligation into pJC70 to yield mutagenesis vector pJC102.

pMY21 – deletion of *mupQ mupS macpD and mmpF*

Primers QDA1F plus QDA1R and QDA2F plus QDA2R were used to amplify 547 bp and 504 bp fragments from the NCIMB10586 chromosome. Primers QDA1R and QDA2F were designed to incorporate an *Xba*I site allowing the two arms to be joined to an in-frame deletion starting in *mupQ* and ending in *mmpF* and cloned using *Eco*RI/*Bam*HI digestion and ligation with pAKE604 digested with *Eco*RI/*Bam*HI to yield mutagenesis vector pMY21.

Construction of expression plasmids

Initial constructions were designed when we predicted *mupO, macpE, mupU, mupV, mupC* and *mupF* to be sufficient for the PAB to PAA conversion. A series of intermediate plasmids were constructed using AT-cloning by the protocol in the pGEM-T Easy manual prior to generation of combinations of genes and then cloning into the broad host-range expression vector pJH10. Primers were designed to include the native NCIMB10586 ribosome binding site (RBS) for each gene. The template DNA for all initial PCRs was the *P. fluorescens* NCIMB10586 chromosome.

pMMG1 – intermediate carrying *mupO*

Primers FmupO and RmupO were used to amplify a 1.4 kb fragment, which was AT-cloned into pGEM-T Easy.

pMMG2 – intermediate carrying *macpE*

Primers FmacpE and RmacpE were used to amplify a 257 bp fragment, which was AT-cloned into pGEM-T Easy.

pMMG12 – intermediate carrying *mupO and macpE*
A 269 bp *macpE*-containing fragment was released by *Age*I/*Xba*I digest of pMMG2, and cloned into pMMG1 digested *Age*I/*Xba*I.

pMMG34 – intermediate carrying *mupU* and *mupV*
Primers FmupUV and RmupUV were used to amplify a 3.6 kb fragment encoding both *mupU* and *mupV*, which was AT-cloned into pGEM-T Easy.

pMMG1234 – intermediate carrying *mupO, macpE, mupU* and *mupV*
The 3.6 kb *mupU* - *mupV* fragment from pMMG34 was released by *Afl*II/*Xba*I digestion and cloned into *Afl*II/*Xba*I-cut pMMG12.

pMMG5 – intermediate carrying *mupC*
Primers FmupC and RmupC were used to amplify a 1.3 kb fragment, which was AT-cloned into pGEM-T Easy.

pMMG6 – intermediate carrying *mupF*
Primers FmupF and RmupF were used to amplify a 1.0 kb fragment, which was AT-cloned into pGEM-T Easy.

pMMG56 – intermediate carrying *mupC* and *mupF*
The 1.0 kb *mupF* fragment from pMMG6 was released by *Avr*II/*Spe*I digestion and cloned into *Avr*II/*Spe*I-cut pMMG5.

pMMG66 – intermediate carrying *mupO, macpE, mupU, mupV, mupC* and *mupF*
The 2.4 kb *mupC* - *mupF* fragment from pMMG56 was released by *HindIII*/*Xba*I digestion and cloned into *Hind*III/*Xba*I-cut pMMG1234.

pMMH6 – expression plasmid carrying *mupO, macpE, mupU, mupV, mupC* and *mupF*
The 7.6 kb fragment from pMMG66 containing all six genes was released by *Kpn*I/*Xba*I digestion and cloned into broad host-range expression vector pJH10 cut with *Kpn*I/*Xba*I.

pJC124 – expression plasmid carrying *mupO, mupP, macpE, mupU, mupV, mupC* and *mupF*
Primers FmupOP and RmupOP were used to amplify a 2.4 kb fragment including genes *mupO* and *mupP*. Primers FmacpE2 and RmacpE2 were used to amplify a 278 bp fragment carrying *macpE*. Primers RmupOP and FmacpE2 were designed to overlap. The products were spliced by overlap PCR with primers FmupOP and RmacpE2, to give a 2.6 kb fragment that was digested with *Kpn*I/*Afl*II and inserted into pMMH6 to replace the *mupO-macpE* segment, yielding pJC124.

pJC132 – expression plasmid carrying *mupO, mupP, macpE, mupU, mupV, mupC, mupF, mupM* and *mupN*
Primers FmupMN and RmupMN were used to amplify a 4.1 kb fragment including genes *mupM* and *mupN*. This product was AT-cloned to pGEM-T Easy. The DNA insert was released by *Xba*I/*Bsa*I digest, and inserted into *Xba*I/*Not*I-digested pJC124 yielding pJC132.

pJC133 – expression plasmid carrying *mupO, mupP, macpE, mupU, mupV, mupC, mupF, mupL, mupM* and *mupN*
Primers FmupLMN and RmupMN were used to amplify a 5.1 kb fragment including genes *mupL, mupM* and *mupN*. This product was AT-cloned to pGEM-T Easy. The DNA insert was released by *Xba*I/*Bsa*I digest and inserted into *Xba*I/*Not*I-digested pJC124 yielding pJC133.

pJC134 – expression plasmid pJC133 with point mutation H256A in MupL
Primers FmupLMN and RmupL-H256A were used to amplify an 809 bp N-terminal segment of *mupL* while primers FmupL-H256A and RmupMN were used to amplify a 4.3 kb fragment with the C-terminal part part of *mupL* plus *mupM* and *mupN*. The overlap in primers FmupL-H256A and RmupL-H256A included the *mupL* H256A mutation (DNA codon CAC to GCC) allowing overlap PCR using outer primers FmupLMN and RmupMN, to generate a 5.1 kb fragment including *mupL*H256A, *mupM*

and *mupN*. This product was AT-cloned into pGEM-T Easy and then released by *Xba*I/*Bsa*I digest before insertion into *Xba*I/*Not*I-digested pJC124 yielding pJC134.

Supporting Information

Figure S1. Genetic map of the mupirocin cluster

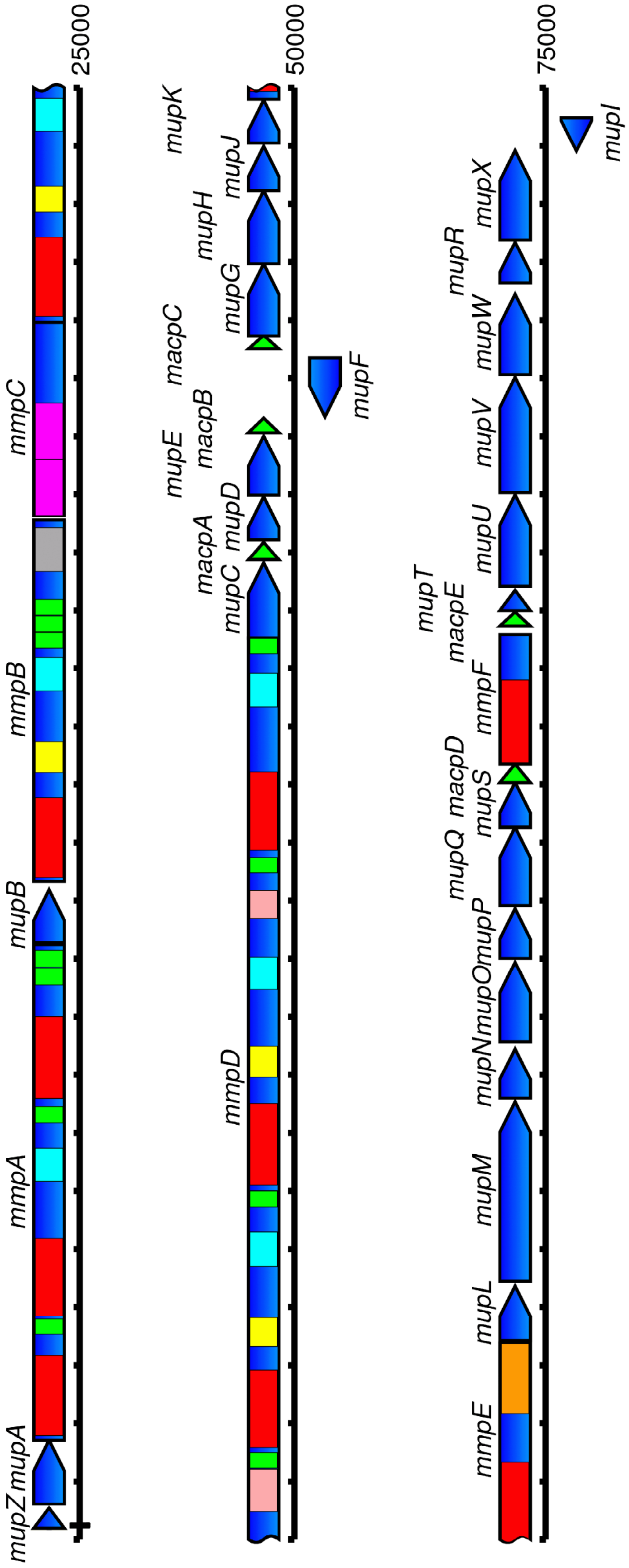

Figure S2. XylE assay: the crude PA-B extract contains the homoserine lactone product of MupI, and activates the *mupA* promoter in 10586 Δ*mupI xylE::mupA*. XylE catalyses conversion of catechol to the yellow compound 2-hydroxymuconic semialdehyde. Representative plate shown, n=9.

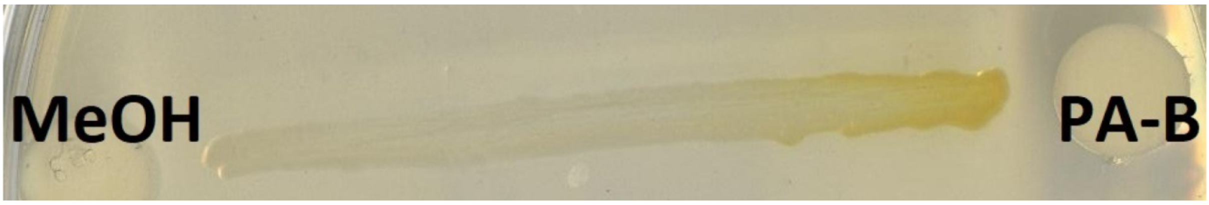

Figure S3. Amino acid alignment of MupV domain 2, His631 is conserved.

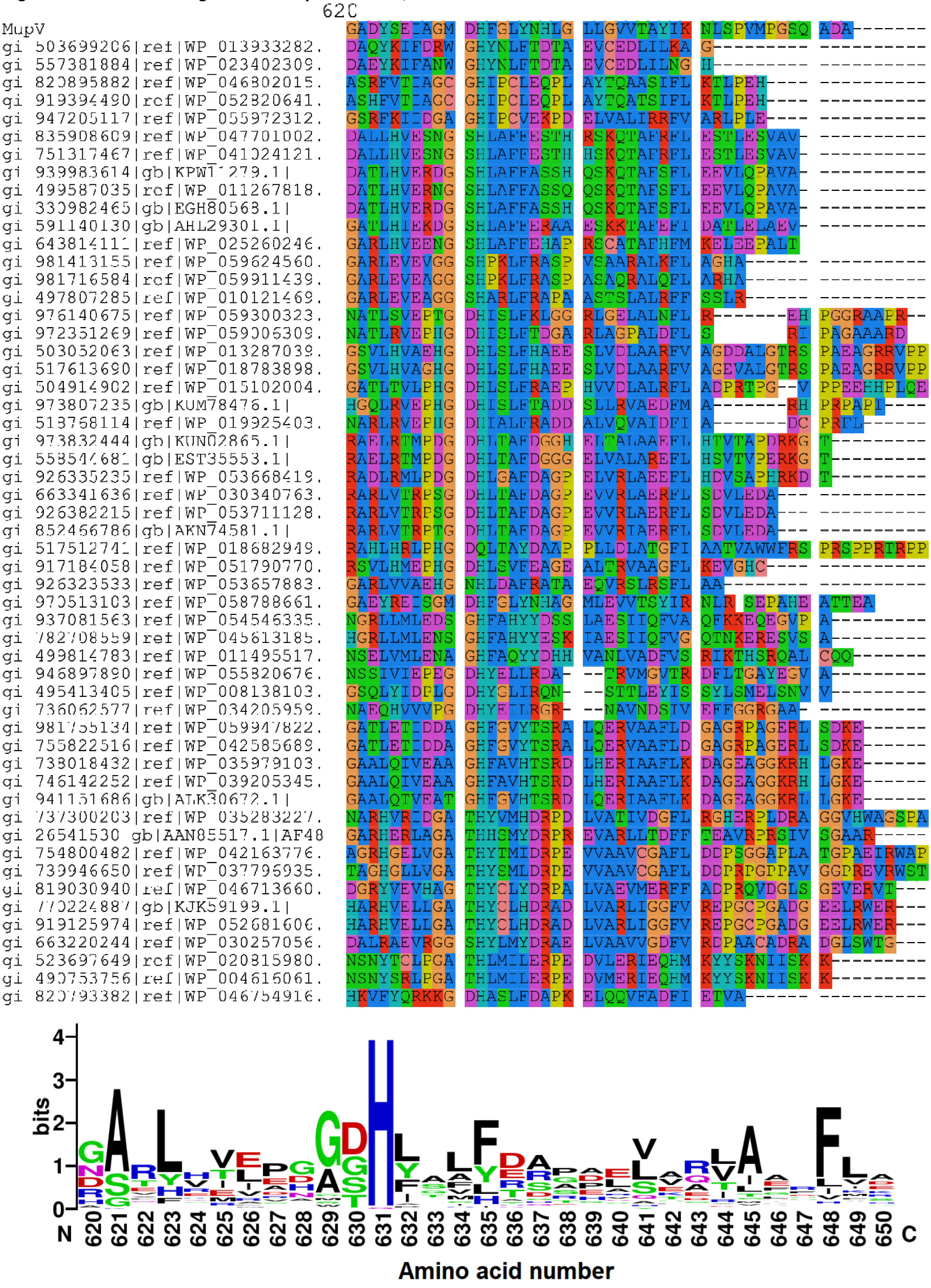

Figure S4. Bioassay activity of 10586 *mupV* point mutants vs *Bacillus subtilis*, n=2.

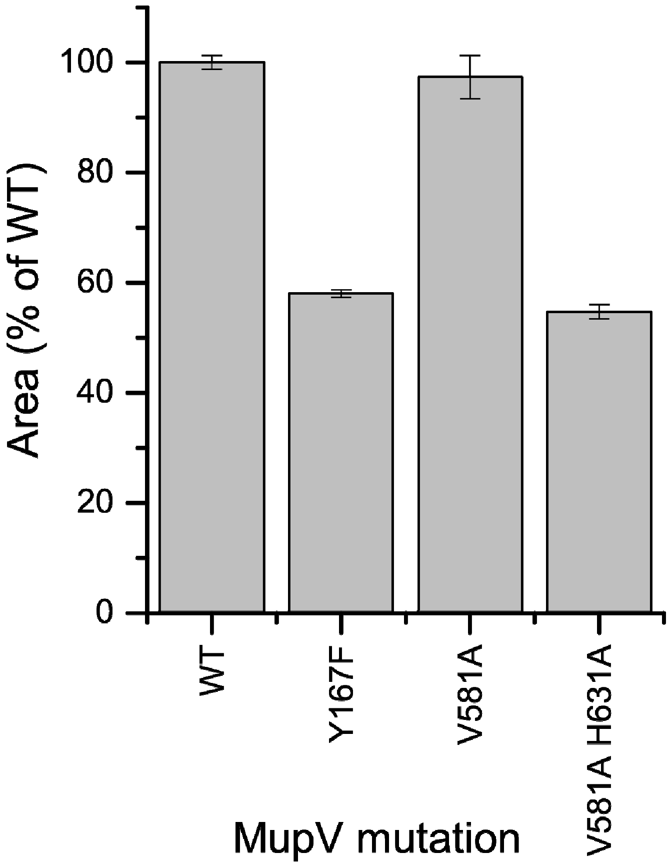

Figure S5. HPLC of 10586 *mupV* point mutants, n=3, metabolite mass confirmed by LC-MS.

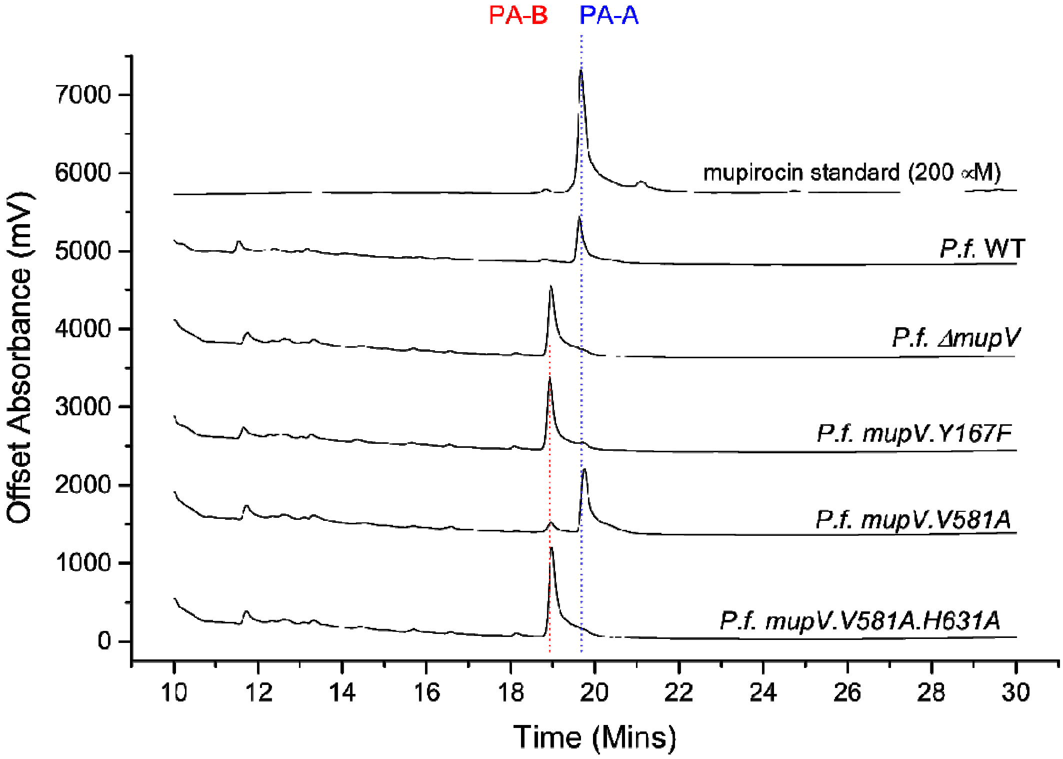

Figure S6. A, LCMS and B, ^{1}H-NMR of 10586 *mupV* point mutants showing that point mutations in MupV domain 1 and domain 2 produce PA-B just like a Δ*mupV* mutant.

S6A.

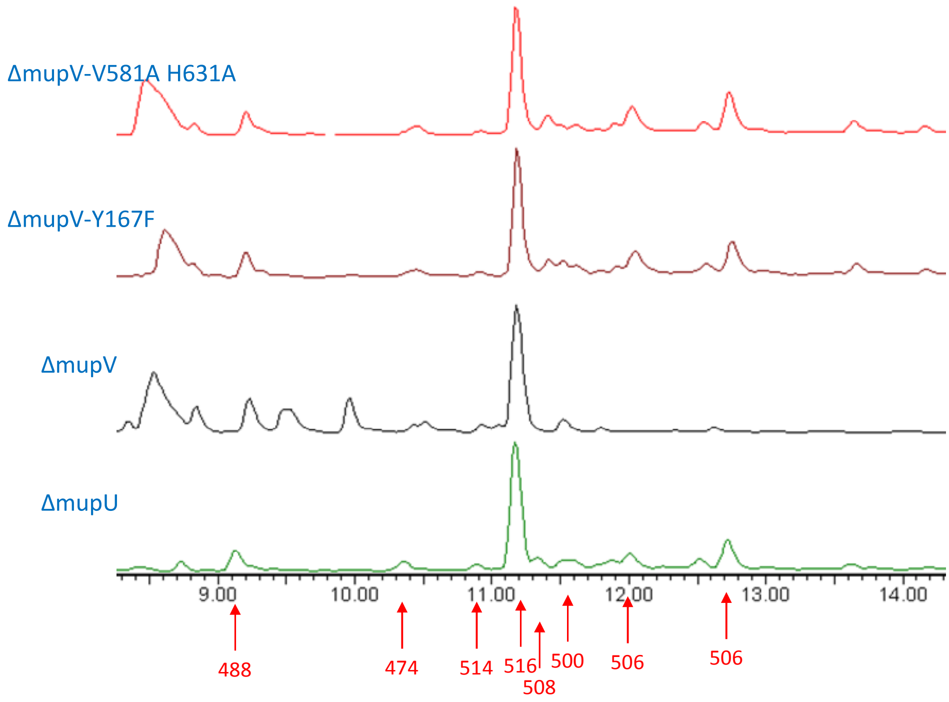

S6B.

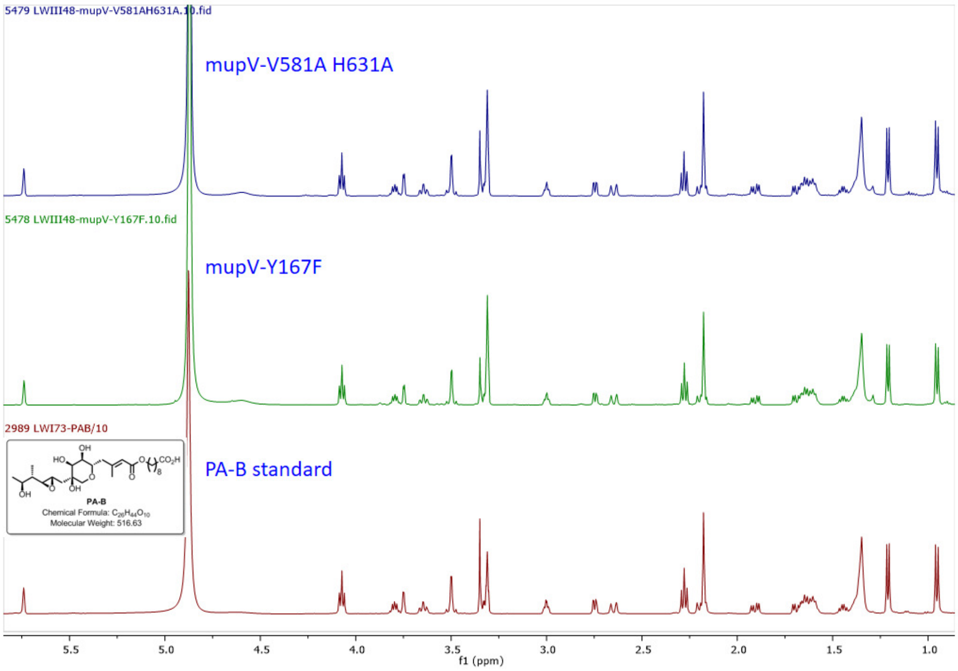
5479 LWIII48-mupV-V581AH631A.10.fid
mupV-V581A H631A
5478 LWIII48-mupV-Y167F.10.fid
mupV-Y167F
2989 LWI73-PAB/10
PA-B standard
PA-B
Chemical Formula: C26H44O10
Molecular Weight: 516.63
f1 (ppm)

Figure S7. HPLC: testing for complementation of 10586 Δ*mupL*, 10586 Δ*mupM* and 10586 Δ*mupN* by pJC132 and pJC133.

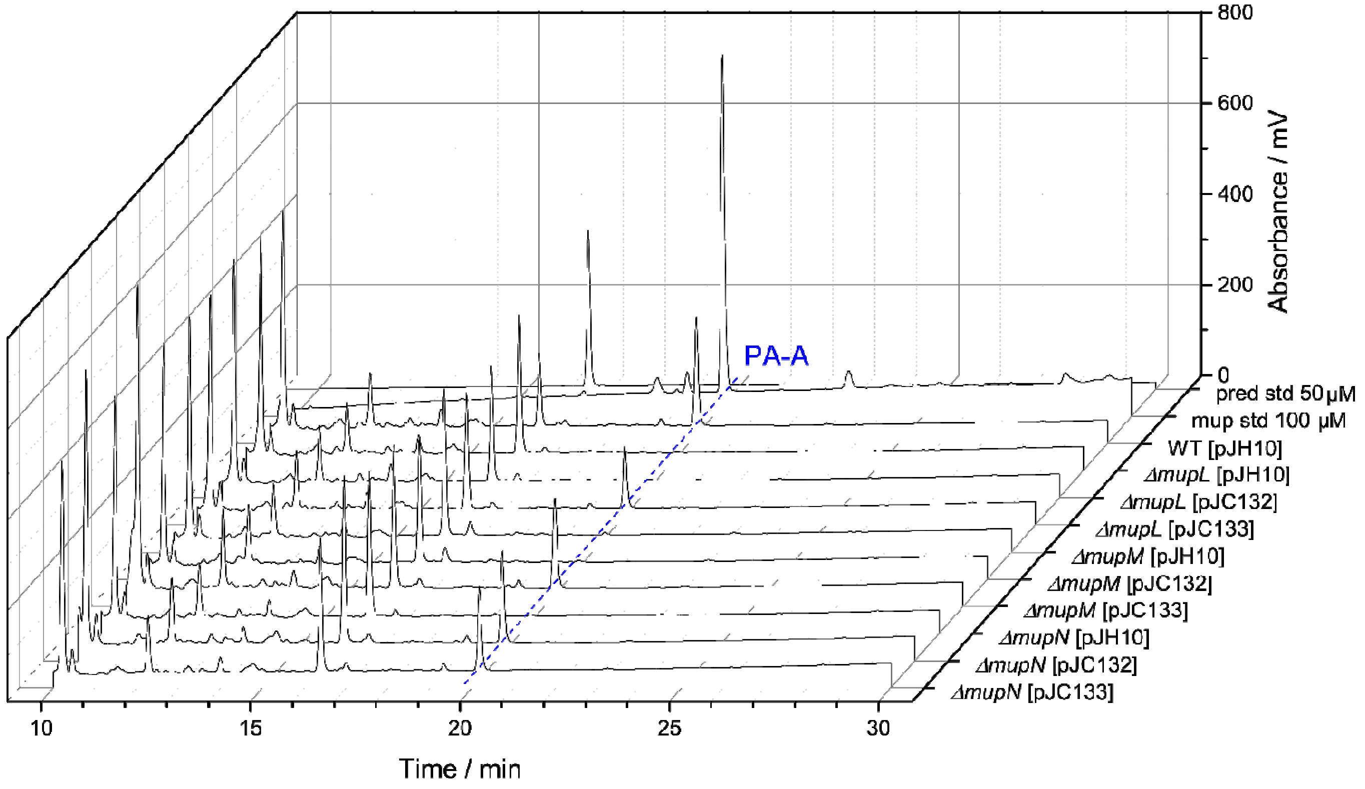

Supplement: Supplementary file 1 — Supplementary Information [file 41598_2018_38038_MOESM1_ESM.pdf]
